# Where are macrophage-tropic viruses?

**DOI:** 10.1186/1758-2652-13-S3-O3

**Published:** 2010-11-04

**Authors:** G Schnell, S Joseph, S Spudich, R Price, R Swanstrom

**Affiliations:** 1 UNC Center For AIDS Research, University of North Carolina at Chapel Hill, Chapel Hill, USA; 2 Department of Neurology, University of California at San Francisco, San Francisco, USA

## Background

Eradication strategies must consider all cellular sources of virus. During the course of infection, HIV-1 can evolve to acquire new cell tropism. We have examined virus in blood and cerebral spinal fluid to identify virus capable of infecting macrophages.

## Methods

Viral env genes were amplified by PCR using limiting template dilution. The amplicons were sequenced and a subset was cloned into an expression vector. The cloned env genes were used to create pseudotyped virus that was then tested for entry into Affinofile cells expressing high and low levels of CD4.

## Results

We examined virus in blood and CSF in eight subjects with advanced HIV disease and with neurocognitive involvement. All eight subjects had highly compartmentalized virus in comparing blood and CSF. In half of the subjects, the virus decayed rapidly in blood and CSF when therapy was initiated, and all env genes tested could infect cells only when there were high levels of CD4, both features indicative of infection of activated T cells in both the blood and central nervous system (CNS). In the other half of the subjects, virus decayed slowly in the CSF, but rapidly in the blood with the initiation of therapy, and the virus in the CSF could infect cells with low levels of CD4, indicative of the presence of macrophage-tropic virus in the CNS. However, the majority of the virus in the blood retained the requirement for high levels of CD4 for infection.

## Conclusions

Macrophage-tropic viruses evolve late in disease and are compartmentalized in the CNS and perhaps elsewhere. They are produced from cells with a long half-life compared with activated T cells. Finally, they are poorly represented in the blood, even when they predominate in the CNS.

